# Echinococcal Disease of the Liver in Pregnancy

**DOI:** 10.1155/1990/93620

**Published:** 1990

**Authors:** John P. Crow, Michael Larry, Elio G. Vento, Richard A. Prinz

**Affiliations:** 1 Loyola University Stritch School of Medicine Departments of Surgery and Obstetrics and Gynecology First Avenue Maywood IL 60153 USA; 2 Loyola University Medical Center Room 2668 First Ave. Maywood Bldg. 102 2160 S. Illinois 60153 USA

## Abstract

Although echinococcal disease of the liver is common in some parts of the world, it is rarely seen in the United States. Even from endemic areas, few reports deal with its treatment during pregnancy. A 25 year old 18 week pregnant Italian woman presented with pruritis and right upper quadrant pain. Ultrasound revealed a cystic lesion in the left lobe of the liver. The cyst was treated by operative insertion of hypertonic saline and capsulorrhaphy. The patient and her fetus had no postoperative complications. We believe that the pregnant patient with symptomatic hepatic echinococcal disease should be treated operatively and that insertion of hypertonic saline and capsulorrhaphy is the safest and most effective technique for both the mother and fetus.


*HPB Surgery* 1990, Vol. 2 pp. 115–119
Reprints available directly from the publisher
Photocopying permitted by license only

© 1990 Harwood Academic Publishers GmbH
Printed in the United Kingdom

### ECHINOCOCCAL DISEASE OF THE LIVER IN PREGNANCY

JOHN P. CROW, MICHAEL LARRY,
ELIO G. VENTO and RICHARD A. PRINZ*

*Loyola University Stritch School of Medicine Departments of Surgery and Obstetrics and Gynecology, 2160 S. First Avenue Maywood, IL 60153*

*(Received 25 February 1989)*

Although echinococcal disease of the liver is common in some parts of the world, it is rarely seen in the United States. Even from endemic areas, few reports deal with its treatment during pregnancy. A 25 year old 18 week pregnant Italian woman presented with pruritis and right upper quadrant pain. Ultrasound revealed a cystic lesion in the left lobe of the liver. The cyst was treated by operative insertion of hypertonic saline and capsulorrhaphy. The patient and her fetus had no postoperative complications. We believe that the pregnant patient with symptomatic hepatic echinococcal disease should be treated operatively and that insertion of hypertonic saline and capsulorrhaphy is the safest and most effective technique for both the mother and fetus.

KEY WORDS: Echinococcosis, pregnancy, surgical treatment, hypertonic saline, capsulorrhaphy

### INTRODUCTION

Hydatid disease of the liver is rarely seen in modern industrialized areas such as the United States. However, due to the frequency of immigration and the ease of intercontinental travel, echinococcosis must be included in the differential diagnosis of any patient who has had potential exposure and develops abdominal pain, jaundice, or fever of unknown etiology. The ease of overlooking this entity is especially true in pregnancy since there are many other more common causes of these non specific findings during that period. Likewise, there are few reports dealing with the appropriate treatment of the pregnant patient with echinococcal disease of the liver. We present the following patient as a reminder that echinococcal disease of the liver must be included in the differential diagnosis of abdominal pain, jaundice and/or fever occurring in pregnancy and to illustrate that operation is not only indicated, but is also safe and effective treatment for these patients.

### CASE REPORT:

A 25 year old, 18 week pregnant Italian female presented to her obstetrician with complaints of pruritis and intermittent right upper quadrant pain lasting seven days. She described the pain as sharp and nonradiating but exacerbated by eating. The pain

Correspondence: R.A. Prinz, M.D. Loyola University Medical Center Room 2668, Bldg. 102 2160 S. First Ave. Maywood, Illinois 60153.

was occasionally accompanied by nausea, vomiting, and low grade fever (38.0–38.5° C.). She denied recent changes in the color of her skin, urine, or stool. The patient described a similar episode ten years ago. She was diagnosed as having had hepatitis by her physician in Italy and the attack resolved after one month of conservative therapy. Her social history was pertinent for exposure as a child to sheep and cattle on her father's farm in Italy. She specifically denied any exposure to dogs or other carnivores during childhood. She immigrated to the United States eight months prior to admission.

On physical exam, she had a low grade temperature. She did not appear toxic and was in no acute distress. She was mildly icteric. Her lungs were clear to auscultation. Her abdominal exam aside from a palpable uterus revealed right upper quadrant and epigastric tenderness without peritoneal irritation. Her liver span by percussion was 10 cm. There was a fullness in the epigastrium. Rectal exam was noncontributory.

Initial laboratory results included: white blood cell count of 12,500 with 78% segs, 18% bands, and 1% eosinophils; serum alanine aminotransferase of 109 U/l; serum aspartate aminotransferase of 76 U/l; total serum bilirubin of 3.4 mg/dl with a direct serum bilirubin of 1.5 mg/dl; serum alkaline phosphatase of 90 U/l; and serum lactic dehydrogenase of 108 U/l. Her remaining chemistries, chest X-ray, and hepatitis profile were normal. Ultrasound examination of her gallbladder and liver showed a 14 cm. cystic lesion compatible with hydatid disease replacing her left hepatic lobe (Figure 1). She had mild intrahepatic biliary duct dilatation, but her hepatic and common bile ducts were not enlarged. The patient was hospitalized for further evaluation. Echinococcal serology by the ELISA Method was performed revealing a negative

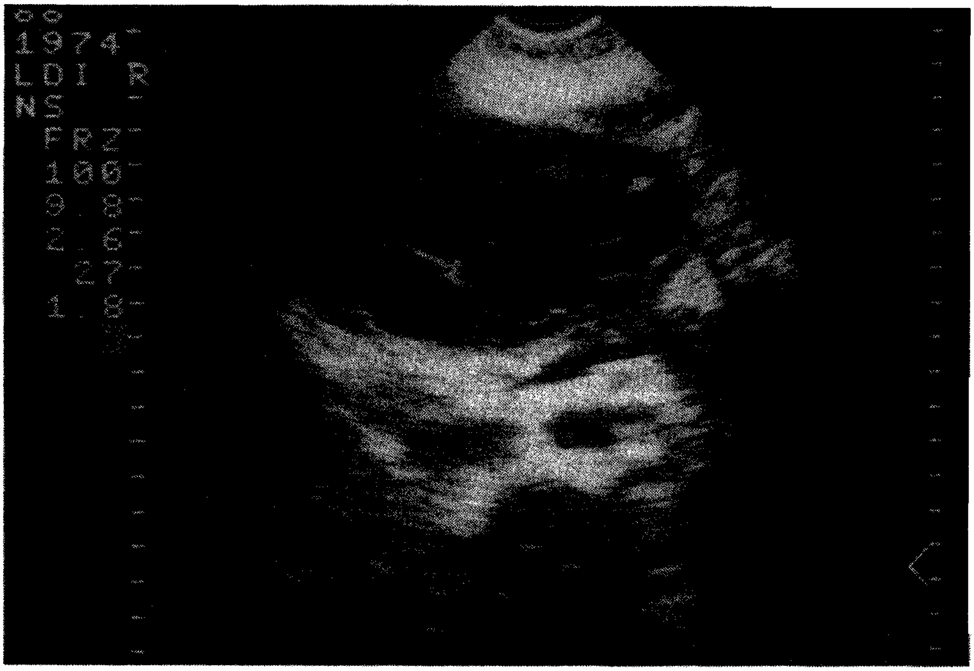

**Figure 1** Hepatic ultrasound demonstrates a 14 cm cystic mass with inner loculations compatible with hydatid disease replacing the left lobe serum IgM titer, a negative serum IgE titer and an IgG titer of 2.7 with normal being less than 2.0. Amebic serology by indirect hemagglutination and immunofluorescent antibody techniques were negative. Repeat liver enzyme studies revealed: serum aspartate aminotransferase was 199 U/dl; serum alanine aminotransferase was 195 U/dl; serum alkaline phosphatase was 126 U/dl; and total serum bilirubin was 1.5 mg/dl. Obstetrical consultation confirmed a viable fetus of 18 weeks gestation.

A decision to operate was made because of the liver function abnormalities and the possibility of rupture during pregnancy and parturition. At operation, a hydatid cyst was found involving the entire left hepatic lobe without evidence of extrahepatic or biliary tract rupture or spread. Isolation of the cyst was accomplished by a technique used by Cohen and co-workers[1]. Laparotomy pads soaked in hypertonic saline were used to pack off and protect the peritoneal cavity. A sterile plastic drape with a central hole was sutured directly to the most superior aspect of the cyst wall. All manipulation of the cyst was performed through this central opening. Sterilization was performed by injecting a 15% saline solution into the cyst cavity. The cyst contents were carefully evacuated and the inner layer of the cyst was debrided. Cyst capsulorrhaphy was performed. Pathologic examination of the aspirate revealed brood capsules and hooklets consistent with *Echinococcus granulosus*. The postoperative course was unremarkable. Fetal viability was confirmed throughout the postoperative period. She was discharged on the fifth postoperative day. Five and a half months later, she was readmitted to hospital for a normal vaginal delivery of a 7lb. 8oz. girl. They were both discharged in good health.

### DISCUSSION:

Hydatid disease of the liver is caused by the dog tapeworm, *Echinococcus granulosus*. This parasite is rarely found in societies with modern slaughterhouses and stray animal control. Man is an intermediate host for echinococcus while dogs, foxes and wolves are definitive hosts. Through contamination with vegetables or oro-fecal contact, intermediate hosts which also include cattle, sheep, swine and caribou are infected with the parasite in the form of a cyst. Cyst capsules are dissolved in the alkaline environment of the duodenum, releasing larvae into the portal blood. The larvae may then form a hydatid cyst. These develop most commonly in the liver and are seen in approximately 70% of patients. As the cyst enlarges, daughter cysts and more importantly, brood capsules are formed. These capsules contain scolices. Each scolex is capable of becoming a new tapeworm in the intestine of the definitive host, thus completing the life cycle of the parasite.

Patients with hydatid disease of the liver may present with a range of symptoms from vague abdominal pain to sepsis from a hepatic abscess. Physical examination may be normal or reveal fever, jaundice, or an abdominal mass. At least one liver enzyme study is elevated in 50% of patients. Eosinophilia although nonspecific, is seen in 25–50% of patients. Serology testing is more specific. In the Casoni skin test, hydatid fluid was injected intradermally and the development of an urticarial papule indicated a positive result. However, this test may cause anaphylaxis, and will be positive in carcinomatosis, leishmaniasis, and teniasis. Newer tests include indirect hemagglutination with an 87% sensitivity but a 10% false positive rate, immunoelectrophoresis and double diffusion with 55% sensitivities and no false positives[2]. A combination of these three serologic tests offers the best chance of detecting specific anti-echinococcal antibodies.

Radiography is helpful in identifying the presence and extent of hydatid disease of the liver. Ultrasound is highly specific although CT scanning is more accurate. CT scan was helpful in diagnosing 49 of 50 cases of echinococcal liver cysts reported by de Diego-Choliz and co-workers[3]. Both of these studies will also help delinate the size of the biliary ducts. Intrabiliary rupture is seen in 10–15% of cases. Ultrasound was obtained in our pregnant patient because it avoids the radiation exposure to the fetus that occurs with CT scanning.

Currently, surgery is the most effective therapy for eradicating the parasite. Three goals must be met with any surgical treatment: death of the parasite; prevention of complications; and obliteration of the residual cavity. Resection is ideal for realizing these goals. However, it can only be safely performed in approximately 10% of cases. Sterilization of the cavity with a variety of scolicidal agents is another option. Hypertonic saline is the agent currently recommended. Formalin, widely used in the past can cause serious morbidity and mortality, and should no longer be used. Once the cyst is sterile and the cyst contents aspirated, the hydatid cyst must be opened and debrided. It is important to remove the innermost layer, the germinal lining, to prevent recurrence. It is equally important to prevent spillage of the cyst contents to prevent potentially fatal anaphylaxis and recurrence.

How to handle the residual cavity is the most controversial issue with this disease. Our method utilized cyst capsulorrhaphy as decribed by Cohen and colleagues[1]. Langer and co-workers[4] from the same group reported a series of 18 uncomplicated cysts in which all 12 patients handled by this technique had a successful outcome with only a 12% complication rate. Five of the remaining six uncomplicated cysts were drained externally with an 80% complication rate. Complications included wound infection, hepatic abscess, intra-abdominal abscess, bowel obstruction, or prolonged tube drainage. Ekram[5] has also reported success with cyst capsulorrhaphy in 21 patients.

Other methods of cyst management include omentoplasty and capitonnage. Pitt and co-workers[6] recommend packing an uncomplicated cyst cavity with the omentum. Capitonnage i.e. obliteration of the cavity by multiple internal sutures is recommended by authors from the Middle East[7]. Marsupialization and external drainage are methods used with complicated cysts[8]. Intrabiliary rupture necessitates choledochotomy at the time of operation to remove hydatid debris from the biliary tree[9].

There is limited information on echinococcal disease presenting in pregnancy. Bickers[10] and Semchyshyn[11] reported pregnant patients with pelvic hydatid disease and recommended excisional therapy. Their patients did not have liver disease, and no guidelines exist in the literature for the treatment of a pregnant patient with hepatic echinococcosis. Operation was recommended in our patient because she was symptomatic with low grade fever and elevation of liver function studies. In addition to excluding intrabiliary communication and hepatic abscess, we believe operation was indicated to prevent possible intraperitoneal rupture of the large cyst during parturition. At operation, excision of the large hepatic cyst was not a reasonable alternative because potential blood loss would increase the risk of morbidity and mortality to both the patient and fetus. Cyst capsulorrhaphy and insertion of hypertonic saline was chosen as the most direct and safe method of eliminating the cyst in this unusual circumstance.

Medical therapy of echinococcosis has been reported in nonpregnant patients. The antiparasitic agents, mebendazole and albendazole, have multiple side effects, and they have teratogenic potential. In the pregnant patient, we would not recommend medical treatment for hydatid cysts of the liver until further studies can be performed to clarify the efficacy and morbidity of these drugs. If our patient had a small, asymptomatic hepatic cyst with negative seology and a heavily calcified wall, an inactive cyst would be likely. Simple observation would be appropriate in this setting since the chance of rupture or other complication is probably very small. Thus each case must be managed individually depending on the status of the mother, fetus and hydatid cyst.

### *References*

1. Cohen, Z. *et al.* (1976) Surgical Treatment of Hydatid Disease of the Liver. The *Canadian Journal of Surgery* **19**, 416–20.
2. Chemtai, A.K. *et al.* (1981) Evaluation of Five Immunodiagnostic Techniques in Echinococcosis Patients. *Bulletin of the World Health Organization* **59**, 767–72.
3. de Diego-Choliz, J. *et al*(1982) Computed Tomography in Hepatic Echinococcosis. *Am J Rad* **139**, 699–702.
4. Langer, B.C. *et al.* (1984) Diagnosis and Management of Hydatid Disease of the Liver: A 15 Year North American Experience. *Ann Surg* **199**, 412–17.
5. Ekrami, Y. (1976) Surgical Treatment of Hydatid Disease of the Liver. *Arch Surg* **111**, 1350–2.
6. Pitt, H.A. *et al.* (1986) Management of Hepatic Echinococcosis in Southern California. *Am J Surg* **152**, 110–15.
7. Dugalic, D. *et al.* (1982) Operative Procedures and Management of Liver Hydatidoses. *World J Surg* **6**, 115–18.
8. Sayek, I., Yalin, R., Sanac, Y. (1980) Surgical Treatment of Hydatid Disease of the.Liver. *Arch Surg* **115**, 847–50.
9. Lygidakis, N.J. (1983) Diagnosis and Treatment of Intrabiliary Rupture of Hydatid Cyst of the Liver. *Arch Surg* **118**, 1186–89.
10. Bickers, W.M. (1970) Hydatid Disease of the Female Pelvis. *Am J O & B* **107**, 477–83.
11. Semchyshyn, S.: Echinococcus Discovered During Pregnancy. *Am J O&B* (1974); **118**, 283–4.

*(Accepted by John L. Cameron 10 April 1989)*

